# Dihydroquercetin-Loaded Liposomes Change Fibrous Tissue Distribution in the Bleomycin-Induced Fibrosis Model

**DOI:** 10.32607/actanaturae.27440

**Published:** 2024

**Authors:** E. V. Ivanov, M. R. Akhmetshina, A. R. Gizatulina, M. V. Gulyaev, O. S. Pavlova, Y. A. Pirogov, S. A. Gavrilova

**Affiliations:** Faculty of Medicine, Lomonosov Moscow State University, Moscow, 119991 Russian Federation; Faculty of Physics, Lomonosov Moscow State University, Moscow, 119991 Russian Federation

**Keywords:** dihydroquercetin, bleomycin, liposomes, antioxidants, pulmonary fibrosis, 1H MRI

## Abstract

The effects of the antioxidant dihydroquercetin (DHQ) were studied in a model of pulmonary fibrosis. DHQ penetration into the lesion was facilitated by encapsulation into liposomes. Pulmonary fibrosis was modeled in rats by intratracheal injection of bleomycin. For the first 7 days, the rats in the treatment group received a liposomal emulsion with DHQ, while in the comparator group rats received saline. In the control group, intact rats did not receive any exposure. Thirty days after the initiation, lung function and the pathological lesion volume were assessed by 7T 1H MRI and the lungs were taken for histologic examination. The proportion of fibrous tissue was counted by Masson’s trichrome staining. Both experimental groups were characterized by a significant functional pulmonary deficiency, with low mortality and a small lesion area. In the rats treated with DHQ, the distribution of fibrous tissue was significantly altered. Significantly more fibrous tissue was found in the center of the lesion, while significantly less was in the interstitial space of alveoli. Lung density at the same time was lower in the treated lungs. Dihydroquercetin encapsulated in liposomes affects the mechanisms of bleomycin-induced pulmonary fibrosis progression in rats. While accelerated fibrosis of the lesion can restrict inflammatory processes, delayed fibrosis of the interstitium can further improve the functional state of the lungs.

## INTRODUCTION

Pulmonary fibrosis poses a significant healthcare threat, even more so now after the COVID-19 pandemic. Numerous studies have demonstrated that serious COVID-19 cases lead to pulmonary fibrosis [1]. Severe coronaviral fibrosis causes persistent respiratory insufficiency and long-term impairment. Fibrotic lesions could occur after severe pneumonia caused by other pathogens, most often viruses [2, 3]. The most severe interstitial lung disease, idiopathic pulmonary fibrosis (IPF), can be treated by numerous anti-inflammatory and antiproliferative drugs such as glucocorticoids, azathioprine, cyclophosphamide, mycophenolate mofetil, and some novel antifibrotic agents like nintedanib and pirfenidone [4, 5]. While the latter agents have shown themselves to increase progression- free survival for three to five years and reduce annual mortality, they are still insufficient when it comes to preventing IPF progression in the course of a lifetime.

Pulmonary alveoli consist of a thin alveolocyte layer vulnerable to different cellular damage sources. During the infectious process, leukocytes secrete numerous substances damaging bacteria, infected, and healthy cells. Among other sources of damage there are reactive oxygen species (ROS) produced by neutrophils and macrophages. Aside from the direct cellular damage caused by lipid peroxidation and DNA oxidation, ROS can damage the surfactant layer and basal membranes, thus subsequently impairing lung repair [1, 2]. Antioxidant administration is favorable in terms of simplicity, safety, and availability. Multiple studies have been conducted to test antioxidant effectiveness in preventing, treating, or slowing pulmonary fibrosis progression. Although most antioxidant agents have failed to show a significant impact in preclinical and clinical trials, it remains unclear why they do not work as expected. The reasons include lack of potency, unfavorable pathway of administration and delivery, as well as unwanted disruption of ROS-related regulatory pathways [3, 4]. There are multiple mechanisms via which antioxidants can prove beneficial in lung fibrosis. Antioxidant resveratrol alleviates fibrosis in rodents by Smad and Smad7 expression inhibition, reduces lung fibroblasts proliferation and differentiation, and reduces collagen deposition [5].

One of the most promising antioxidants is quercetin and its derivatives such as dihydroquercetin (DHQ). In several experimental studies, quercetin has shown an ability to attenuate lung fibrosis [6, 7]. Yuan et al. demonstrated that DHQ markedly attenuates a SiO_2_-induced lung inflammation and fibrosis in mice [8]. Impellizzeri et al. found similar effects of quercetin in the bleomycin-induced fibrosis model [9]. One of the major limitations in using quercetin or DHQ alone in the treatment of pulmonary fibrosis is a delivery problem, since the substance is poorly soluble. Targeted delivery of antioxidants is also important as they could be consumed by various other tissues. A delivery system could be used to overcome those issues. Liposomes were found to passively target inflammatory sites, since they are characterized by a leaky vasculature [10]. Liposomes have been evaluated as a delivery platform to treat pulmonary fibrosis with different loaded drugs *in vivo*. Liu et al. have shown successful delivery of Nrf2 blockers in ROSsensitive liposomes through inhalation [11]. Li et al. reported the effectiveness of neutral liposomes loaded with antifibrotic drugs through inhalation in an established pulmonary fibrosis model [12]. Other researchers have used loaded liposomes to target pulmonary fibrosis through systemic parenteral infusions with RNA-based agents [13]. While the oral route of delivery has not been studied as extensively for liposomecarried antifibrotics, it remains valid, since many studies have shown that liposomes could be taken into the lymphatic system from the small intestine [14, 15, 16, 17].

In this study, we chose dihydroquercetin stabilized in liposomes (Flamena emulsion, research company ‘Flamena’, Russia) as an intervention means to prevent pulmonary fibrosis progression in bleomycin-induced pulmonary fibrosis in rats, since this formulation has previously shown promising results in other pathologies thanks to its anti-inflammatory and antioxidant properties [18, 19, 20, 21].

## EXPERIMENTAL

**Animal Handling**

The authors followed the European Convention for the Protection of Vertebrate Animals used for Experimental and other Scientific Purposes and local rules for conducting scientific research with laboratory animals. The experiment was approved by the local bioethics committee at its meeting held on February 9, 2023, Protocol No. 2. Male Wistar rats with a body weight ranging from 200 to 250 g were procured from a conventional breeding facility in the Institute of Medical and Biological Problems (Moscow, Russia). A total of 30 rats were enrolled, 10 in each group. The experiment was launched after an initial 2-week period of acclimatization and handling. Throughout the experiment, the animals were housed in a certified vivarium with a 12-hour day/night cycle and were provided with ad libitum access to standard chow and water. Upon arrival, the animals were separated into groups based on their body weight and placed in standard T3 cages.

**Fibrosis model**

Thirty animals were distributed into three groups, ten animals per group: Flamena, Saline, and Intact. The rats in the Intact group were not subjected to any interventions throughout the study as a healthy control group. In the two experimental (fibrosis) groups, the animals received an intratracheal bleomycin injection (7.5 mg/kg) under isoflurane anesthesia; dosage and route of administration were chosen according to those used in refs. [22, 23]. The bleomycin solution was administered by injection to ensure accurate administration and dosage, as well as uniform delivery of the substance to the lungs. The skin of anesthetized rats was disinfected, and a short incision was made over the cricoid cartilage of the larynx. The soft tissues were separated with tweezers in such a way as not to cause bleeding and not to affect the thyroid gland. After visualization of the larynx and trachea, the head end of the manipulation table was raised. The required amount of the drug was administered slowly using a syringe. The dose and route of administration were selected according to the literature. After insertion, the tissue was sutured with atraumatic suture material and the skin was treated with an antiseptic. A 30-day period was deemed long enough to ensure that a pathological lesion of sufficient size will form and be small enough to avoid possible spontaneous healing in the long term.

**Treatment**

Twenty-four hours after bleomycin administration, we started treatment with equal amounts of liposome-encapsulated dihydroquercetin (Flamena emulsion) or sterile saline. According to license documents and published information, Flamena is a phospholipid emulsion which contains 30 mg/mL lecithin, 35 mg/mL glycine, and 4 mg/mL dihydroquercetin [20]. At least one membrane phospholipid is vortexed with water and ethanol to obtain a liposomal phase containing active ingredients (Patent RU 2369383, October 30, 2007). A combined route of administration was chosen to achieve greater exposure. Previously, we had found Flamena to be effective in a rat myocardial infarction model after oral administration [19]. Since other liposomal carriers had been reported to be effective in lung fibrosis as described in the Introduction section, we chose to apply both approaches because specific portions of damaged lungs could fail to receive the drug through a single route of administration. The published and digital data indicate that Flamena is safe at various doses and routes of administration [21]. A prewarmed to room temperature emulsion (25 mg/kg or 6.25 mL/kg) was administered per os through a gastral tube. The animals were then subjected to 15-min drug inhalation in a 20 L chamber through an ultrasound inhaler (approximately 5 mL of the drug was evaporated per three animals). Double drug administration was continued for 5 days.

**Endpoint**

The mortality rate was assessed until the endpoint. Weight was measured prior to the final procedures. MRI was performed on day 30 under isoflurane anesthesia to assess the lesion size. Under deep over-anesthesia, the rib cage was opened; the heart and lung vessels were perfused with a 1% neutral buffered formalin solution until heart arrest. Lungs were excised and washed in a buffer solution, then they were weighed with a 10 mg precision scale (Sartorius). The lungs were filled with the 1% neutral buffered formalin solution through the trachea and sliced for further histological processing through the basal and upper lobes. Slices with pathological lesions no thicker than 6 mm were immersed in a 4% neutral buffered formalin solution for 36 h.

**MRI**

The study was performed on a 7T MR scanner (BioSpec 70/30 USR; Bruker BioSpin, Ettlingen, Germany) operated with a ParaVision® v.5.1 console and equipped with a 105 mT/m gradient amplitude device. Lung images were recorded using a Birdcage volume radiofrequency coil with an inner diameter of 72 mm. Anesthesia was induced with 4% isoflurane in a chamber, followed by maintenance at 1.5% via a nose mask in 95% O_2_ at a flow rate of 1 L/min. Isoflurane was administered using a vaporizer (Ugo Basile, Italy), while oxygen was supplied by a JAY-10 oxygen concentrator (Longfian Scitech Co. LTD, China).

Lung MR images were acquired using a 3D ultrashort echo time (UTE) pulse sequence with radial kspace filling [24]. The scan parameters were set as follows: scanning area 7 × 7 × 7 cm^3^, scanning matrix 152 × 152 × 152, frequency bandwidth 100 kHz, TE = 18 μs; TR = 8 ms; flip angle = 6°; number of averages = 1; radial projections = 72,231; and polar undersampling = 1. The total acquisition time was 9 min and 38 s.

**Post-processing of the MRI data**

Radial pulse sequences, such as UTE, are less affected by motion artifacts, allowing for lung imaging without the requirement of breath synchronization. As a result, the lung MR images are averaged over the respiratory cycle. Furthermore, it is possible to retrospectively gate the raw data and generate two images corresponding to the inspiration and expiration respiratory phases. This additional insight enhances the diagnostic capabilities of MRI for evaluating lung conditions and respiratory disorders.

The pulmonary MRI data were processed following the methodology outlined in ref. [25]. The center of the radial k-space is oversampled, and the magnitude of the first point of each collected projection (FID, free induction decay) is modulated by the respiratory process. Consequently, the first points of each projection reflect the respiratory phase. These data can be sorted to produce two new k-spaces with incomplete filling derived from the original k-space: one representing the inspiration phase, and the other one, the expiration phase. The final step involves reconstructing gated data using the iterative sampling density compensation function, followed by resampling onto a Cartesian grid before the fast Fourier transform.

The retrospective gating method was implemented using Python 3.8 and Matlab 2019b (MathWorks, USA), resulting in two sets of MR images corresponding to the inspiration and expiration phases. Since the inspiration phase occurs faster than the expiration phase, 8–10% of the projections correspond to inspiration, while 55–60% correspond to expiration. Consequently, the image quality (signal-to-noise ratio, sharpness, and resolution) of the expiration phase is noticeably higher. Based on this, we used the expiration images to delineate lung masks during exhalation and pathology masks, while the inspiration images were utilized solely for lung masks during inhalation. The lung and pathology masks were manually segmented using the ImageJ software (v.1.51j8, NIH, Bethesda, MD, USA) [26] with the freehand selection tool. From the resulting binary masks, the volumes of inspiration (Vinsp), expiration (V_exp_), and pathology (V_pat_) were calculated by summing the non-zero pixels and multiplying by the voxel resolution. Additionally, the respiratory volume (V_resp_ = V_insp_ – V_exp_), respiratory ratio (V^resp^/V_exp_), and pathology percentage volume (V_pat_/V_exp_) were calculated. The expiratory volume to lung weight ratio, Vexp/m, mL/g, was calculated to approximate lung density.

**Histological processing and staining**

Afterwards, fixation tissues were rinsed with tap water for 2 h and dehydrated with ascending isopropanol solutions, mineral oil, and liquid paraffine. Standard paraffine blocks were embedded and left to rest for at least 24 h. Paraffin-embedded blocks were sliced to 5 µm thick slices on a ThermoFisher S355 rotary microtome. Standard hematoxylin and eosin staining (Leica, USA) was performed for general tissue integrity evaluation. Fibrous tissue collagen distribution was revealed with Masson’s trichrome staining (Biovitrum, Russia).

Slides were analyzed and digitalized using a Zeiss Axio A2 microscope and the Zeiss Zen software. Images were further processed with the ImageJ software. A 27-point scale was developed to analyze the degree of pathological involvement. Nine parameters were assessed with a 4-point scale, from score 0 (normal tissue) to score 3 (unfunctional tissue, total inflammation, abundant fibrosis) in each field of view (FoV): presence of alveoli, alveolar integrity, bronchial integrity, inflammation severity, type of infiltration, necrosis in FoV, presence of fibrous tissue, interstitial fibrosis, and focal fibrosis. Ten random FoVs per slide with ×200 magnification were averaged.

Masson’s stained slides were pictured in three areas: pathological lesion, the nearest alveoli-containing region, and the distant alveoli-containing region. Five FoVs were picked for each area with a ×200 magnification. Blue/red segmentation was performed based on the HSV color scale. Total blue and red areas, and the blue-to-red ratio, were calculated.

**Statistical analysis**

All data processing and analysis were conducted using the Python 3.8 programming language and opensource code libraries. Statistical analyses were performed using the Statsmodels and Scipy libraries. Due to the small sample sizes, non-parametric statistical tests and universal statistical models were employed. The Statsmodels library’s mixed-effects generalized linear model (GLM) was used to analyze time-dependent changes. The Kruskal–allis test was employed for dependent variables, followed by post-hoc multiple comparisons using the Dwass-Steel- Critchlow-Fligner (DSCF) test. Screening correlation analyses were conducted using simple Spearman R calculations. All *p*-values less than 0.05 were considered statistically significant. Data visualization was conducted using boxplots, depicting medians, quartiles, and the minimum/maximum values, with the Matplotlib and Seaborn libraries.

## RESULTS

**Mortality and weight gain**

Throughout the experiment, the mortality rate reached 20% (N = 2) in the Saline group, of which one rat died after final anesthesia application. In the Flamena group, the mortality rate was 10% (N = 1): no significant difference was found with the chisquared test. Since the mortality rate was low, we did not perform a survival analysis of death risk factors.

Weight gain in both groups was significantly reduced compared to that in intact rats. Through the 30 days of the experiment, Intact rats gained 23.93% (IQR 20.42, 28.33) off the baseline weight, while Saline group rats gained 14.65% (IQR -8.55, 22.08,* p *= 0.047), and Flamena group rats gained 11.58% (IQR -1.21, 17.07, *p *= 0.01). The two fibrosis groups did not show significant differences in weight gain (*p *= 0.86).

**Lung weight**

**Fig. 2:**
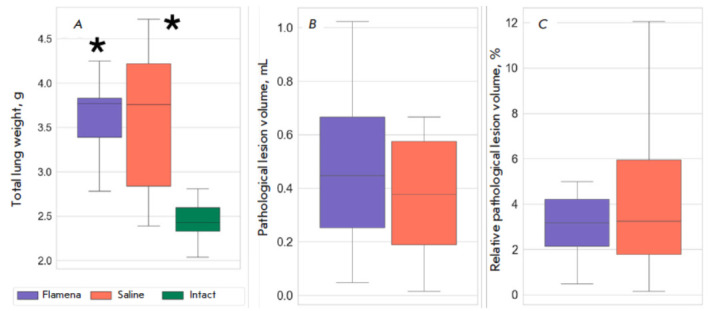
Structural changes in bleomycin-induced fibrosis. (*A*) – Total lung weight, g; (*B*) – absolute pathological lesion volume, ml; (*C*) – relative pathological lesion volume, %. *p  < 0.05 compared to the Intact group

Lungs were weighed gross after the excision. Total lung weight in the fibrosis groups was 1.5 times higher compared to that in intact rats. In the Saline group, it reached 3.76 g (IQR 2.84, 4.22, *p *= 0.001 vs. the Intact group, *Fig. 2A*); in the Flamena group, it reached 3.77 g (IQR 3.39, 3.83, *p * < 0.001 vs. the Intact group). In the Intact group, the median lung weight was 2.43 g (IQR 2.33, 2.60). Weight values did not differ between the two fibrosis groups (*p* = 0.796).

**MRI results**

**Fig. 1:**
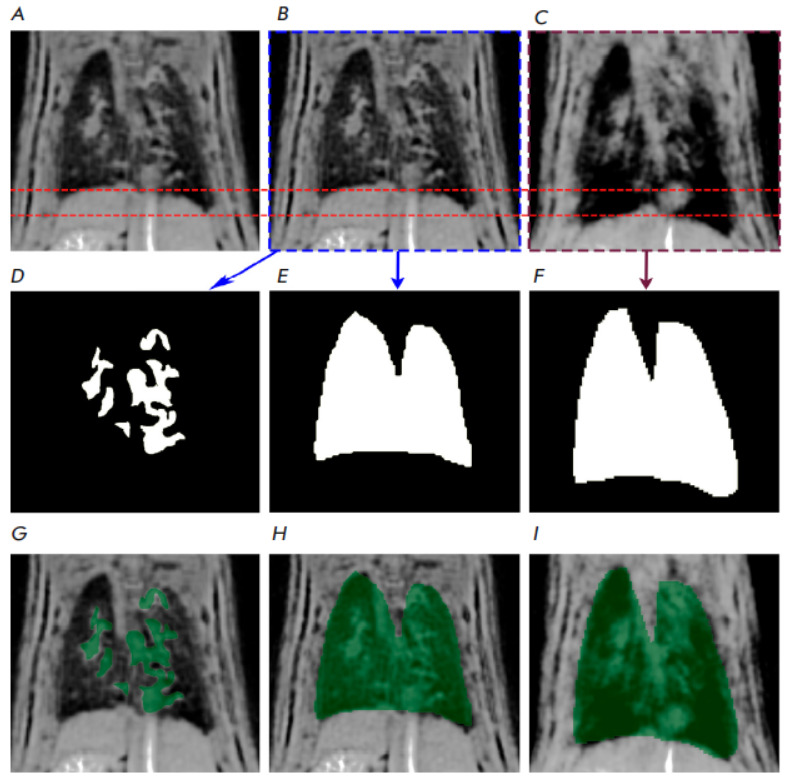
Pulmonary MR images of a randomly selected rat from the Saline group along with the corresponding lung and pathology masks. The data are presented only for one slice. (*A*) – initial MR image of the lungs; (*B*) – image of the expiration phase; (*C*) – image of the inspiration phase; (*D*) – pathology mask; (*E*) – expiration mask; (*F*) – inspiration mask; (*G*) – pathology mask overlaid on the initial MR image of the lungs; (*H*) – expiration mask overlaid on the image of the expiration phase; (*I*) – inspiration mask overlaid on the image of the inspiration phase

The results of MRI data processing for one of the rats are shown in *Fig. 1*.

Two groups did not show statistically significant differences in both the absolute pathological lesion volume (0.38 mL (IQR 0.18, 0.61): in the Saline group, 0.45 mL (IQR 0.25, 0.67), in the Flamena group, *p* = 0.606), and the relative lesion volume (3.24% (IQR 1.77, 6.04) in the Saline group, 3.18% (IQR 2.14, 4.22), *p* = 0.96, *Fig. 2B and 2C*).

**Table 1:** Functional parameters of the lungs for the various groups of animals used in the experiment

Group animals	V_exp_, mL		V_insp_, mL=""		V_resp_ /V_exp_, arb.="" units		V_resp_ /V_exp_, %	
	ME	IQR	ME	IQR	ME	IQR	ME	IQR
Intact	9.76	9.49–10.20	7.54	7.04–8.16	0.31*	0.27–0.35*	-	-
Saline	13.02	11.58–14.49	10.45	9.39–11.75	0.23*	0.15–0.29*	3.61	1.77–6.04
Flamena®	14.27	13.59–15.75	11.78	11.18–13.31	0.17*	0.09–0.21*	3.52	2.11–4.61

Note. ME – median values, IQR – interquartile range. *p $lt; 0.05.

**Fig. 3:**
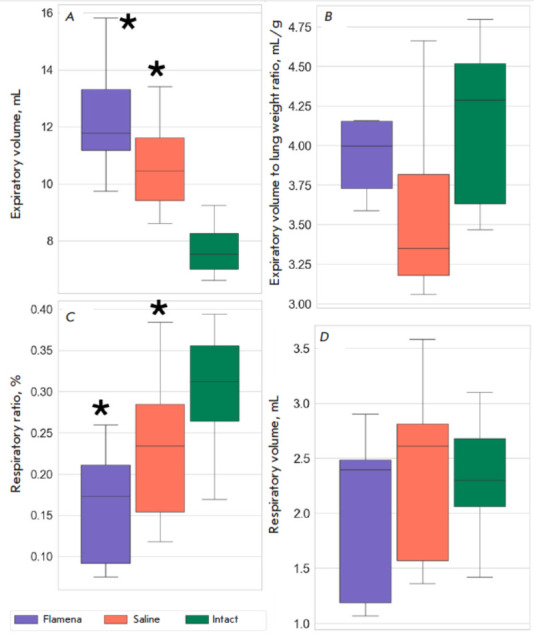
Functional lung changes in bleomycin-induced fibrosis. (*A*) – Expiratory volume, mL; (*B*) – expiratory volume to lung weight ratio, mL/g; (*C*) – respiratory ratio, %; (*D*) – respiratory volume, mL. *p  < 0.05 compared to the Intact group

The expiratory (V_exp_) and inspiratory volumes (Vinsp) were higher in the fibrosis groups compared to the Intact group (*Table 1*). While the respiratory ratio (Vresp/Vexp) was significantly lower in the fibrosis groups compared to the Intact group, the respiratory volume (Vresp) did not differ, probably because of the uncontrolled breath ratio during MRI scanning, which led to a large spread of this value (*Fig. 3*). Among all the characteristics, the expiratory volume (V_exp_) was the most distinct in the Flamena and Saline groups, with *p *= 0.074 (*Fig. 3A*). Since the lung weight was much higher in the fibrosis groups, we also calculated the expiratory volume (Vexp) to lung weight ratio, which was used as a surrogate for a lung density measurement (*Fig. 3B*). While in the Saline group lungs had less volume per unit of weight compared to Intact rats (*p *= 0.027), indicating higher density, no statistically significant differences were found in the Flamena and Intact groups (*p *= 0.387). The median values in the two fibrosis groups almost reached significant differences, with *p *= 0.059.

**Lung histology**

**Fig. 4:**
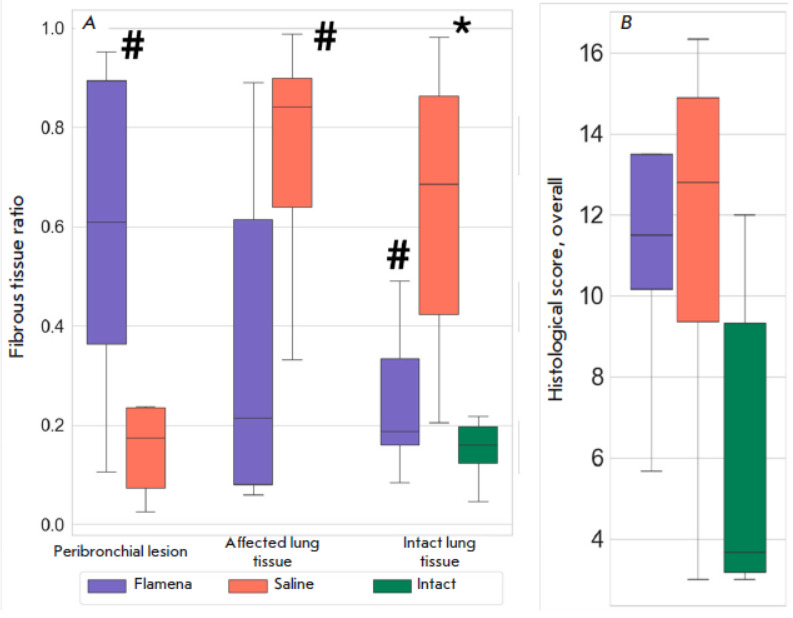
Assessment of histological integrity. (*A*) – The total histological integrity score; (*B*) – blue-to-red pixel area ratio. *p  < 0.05 compared to the Intact group, #*p * < 0.05 compared to the Saline group

Histology analysis was performed with slices obtained from the lobe base, where larger lesions were found. H&E-stained slices were evaluated semi-numerically using a 27-point scale. While the analysis was performed with averaged random field of views, the overall score did not differ in the two groups, with a median of 12.80 points (IQR 9.36, 14.88) in the Saline group and a median of 11.50 points (IQR 10.17, 13.50,* p *= 0.918) in the Flamena group (*Fig. 4A*).

**Fig. 5:**
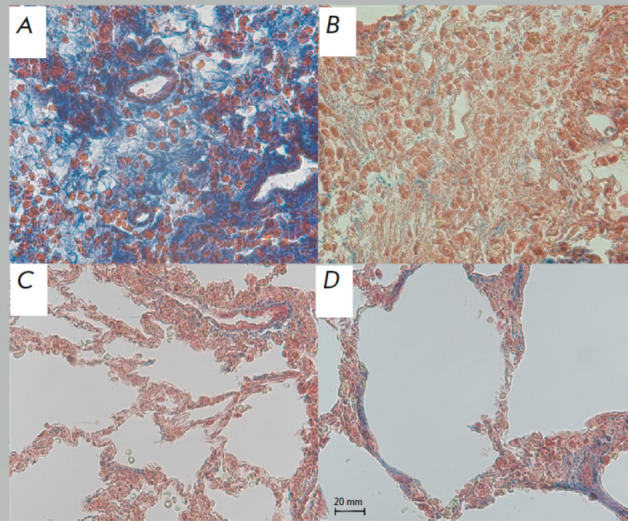
Lung histology revealing collagen deposition patterns. (*A*) – Pathological lesion, the Flamena group; (*B*) – pathological lesion, the Saline group; (*C*) – distant lung tissue, the Flamena group; (*D*) – distant lung tissue, the Saline group. Masson’s trichrome staining, ×200 magnification

Masson’s staining revealed that collagen bundles were distributed differently in the Flamena and Saline groups. Fibrosis inside the pathological lesion represented a finished inflammatory phase, and in the Flamena group, intense collagen staining was observed. Despite the same lesion size, in most slides in the Saline group, lesion did not show collagen deposition as much, with more necrosis, inflammation, and even Masson-negative fibers. The blue/red ratio in the pathological lesion was 0.61 (IQR 0.35, 0.92) in the Flamena group and 0.16 (IQR 0.07, 0.23) in the Saline group (*p* = 0.006, *Fig. 4B*). In the most affected lung tissue, the blue/red ratio was significantly higher in the Saline group, with 0.21 (IQR 0.12, 0.62) vs. 0.72 (IQR 0.64, 0.92), *p *= 0.01. In the most intact lung tissue, more intense interstitial collagen staining also was revealed in the Saline group, with a ratio of 0.71 (IQR 0.42, 0.85) vs. 0.19 (IQR 0.16, 0.34),* p *= 0.01. Intact tissue collagen staining was comparable with that in the Flamena group (0.17 (IQR 0.12, 0.2), *p *= 0.27), Interstitial fibrous tissue deposition was usually present along with interstitial space thickening and intense inflammation (*Fig. 5*).

## DISCUSSION

Antioxidant treatment for pulmonary fibrosis has been evaluated in many studies with encouraging, but still not definitive, results. In our study, we evaluated dihydroquercetin stabilized in liposomes in the rat bleomycin-induced fibrosis model. The latter is one of the best studied and widely used experimental models of pulmonary fibrosis; therefore, its features are well-known. Liposomal carriage improves both DHQ stability and potency; hence, we expected to find prominent treatment effects. Liposomes also ensure selectivity towards inflammatory sites [10]. The administration regimen was semi-arbitrary; higher cumulative dosage or extended period of treatment potentially could lead to better results. Since Flamena has previously been found to be effective in the rat myocardial ischemia/reperfusion model, we expected increased exposure through the combined regimen [19]. We chose to treat rats 24 h after the bleomycin injection, emulating preventive antifibrotic treatment in a severe pulmonary infection like COVID-19.

In our study, the overall mortality rate was low and any survival statistical changes were not possible. We used 7T MRI as one of the best options to study the lesion size and lung excursion. Pathological lesions in most cases did not occupy large parts of their lungs. Hence, it was hard for any type of intervention to reveal drastic results. However, as the lung weight and its density changed, there was a significant degree of pulmonary disfunction. Rats treated with Flamena tended to have less dense lungs, which is an important sign of a less severe inflammation and fibrosis.

There are many varieties of bleomycin-induced fibrosis models, and they are accompanied by various degrees of pathological involvement. Single-dose intratracheal administration usually leads to comparable results. Over one month, no mortality at all or a low mortality rate is usually observed in male rats [27]. While most of the studies provide no quantitative lesion volume measurements, those that do report about 3% of fibrous tissue prevalence, same as in our study [28, 29]. Histological changes in the lung tissues identified by us were similar to those reported in the literature: widespread inflammatory infiltration, necrotic foci, and bundles of collagen fibers. Alveoli near pathological lesions had thicker walls and were usually fused and dysfunctional with damaged walls [30, 31]. According to the literature [22, 27], pulmonary fibrosis in the bleomycin model becomes significant as early as two weeks after the initiation and persists for several months. While fibrosis could be reversed 3 to 6 months after the injection in rodents, it became full blown one month after the injection [32, 33, 34, 35].

In our study, the use of Flamena significantly changed the distribution of fibrous tissue. Interstitial fibrosis is a serious complication that can lead to further decline in lung function, and its signs were less noticeable in the treated group. On the contrary, an increase in the amount of fibrous tissue within the pathological focus is a positive sign, since it indicates a more rapid resolution of inflammatory reactions. Quercetin is a drug that eliminates the signs of aging. It interacts with aging cells, enhances their apoptosis, and reduces the fibrogenic potential. DHQ in its stabilized form is a more powerful antioxidant, and we expected it to be at least as effective as quercetin. DHQ was found to be effective in various diseases but has not been studied as extensively in bleomycin fibrosis. We have shown that DHQ, when delivered in liposomes, can alter the mechanisms of development of bleomycin fibrosis. Although this did not cause significant changes in lung function, differences may be emerge over longer periods.

## CONCLUSIONS

Our study has shown that stabilized DHQ can significantly modify fibrosis and alter its features. Fibrosis within the pathological focus is necessary to prevent the spread of infection and inflammation. Faster remodeling of the lesion may be beneficial and limit excessive inflammation. Progression of interstitial fibrosis in the functional alveoli can lead to severe respiratory impairment.

In a first, we studied the effects of stabilized dihydroquercetin in liposomes on bleomycin-induced pulmonary fibrosis and found that it limited interstitial fibrosis. Further studies with longer duration and different bleomycin administration protocols are needed to determine whether DHA can reduce morbidity and mortality in the long term.

## Abbreviations

ROS: reactive oxygen species
DHQ: dihydroquercetin
IS: impulse sequence
MRI: magnetic resonance imaging
FID: free induction decay
FoV: field of view
HSV: hue, saturation, value
TE: time to echo
TR: repetition time
UTE: ultrashort echo time
